# Metabolic bacterial genes and the construction of high-level composite lineages of life

**DOI:** 10.1016/j.tree.2015.01.001

**Published:** 2015-03

**Authors:** Raphaël Méheust, Philippe Lopez, Eric Bapteste

**Affiliations:** UMR7138 Evolution Paris-Seine, Institut de Biologie Paris-Seine, Université Pierre et Marie Curie, 9 quai saint Bernard, 75005 Paris, France

**Keywords:** evolutionary transition, prokaryotic evolution, tree of life, web of life, lateral gene transfer, eukaryogenesis

## Abstract

Understanding how major organismal lineages originated is fundamental for understanding processes by which life evolved. Major evolutionary transitions, like eukaryogenesis, merging genetic material from distantly related organisms, are rare events, hence difficult ones to explain causally. If most archaeal lineages emerged after massive acquisitions of bacterial genes, a rule however arises: metabolic bacterial genes contributed to all major evolutionary transitions.

Making sense of the origins of major lineages of life, and therefore of the ways by which novel physiologies, ecological systems, and classes of organisms evolved is possibly as important as understanding the origin of species [1]. It could provide fundamental insights about the biology and the intimate make-up of many organisms investigated by microbiologists, ecologists, developmental biologists, geneticists and evolutionary biologists. Recent findings [2–4] proposed that unsuspected major evolutionary transitions occurred amongst prokaryotes, because most archaeal lineages emerged as the result of massive acquisitions of bacterial genes. Therefore, major archaeal lineages would be in part composed of bacterial genes. While the mere discovery of composite archaeal lineages is already thought-provoking, it takes an even greater significance when considered in a broader biological context (Figure 1). This latter comparison unravels a remarkable trend: all major evolutionary transitions leading to novel composite high-level lineages might have benefited from the merging of genetic material from bacteria with genetic material from other sources.

The contribution of bacterial genes to eukaryotic evolution is well-acknowledged [4–6]. Eukaryotes appear as genetic chimera, largely composed of metabolic genes from bacterial origin, while another part of their genomes likely originates from archaea. Eukaryogenesis would have indeed involved (at least) these two kinds of partners: an ancestral bacterial lineage and an ancestral archaeal lineage [4–6]. This dual origin of eukaryotes is without doubt one of the main evolutionary events that occurred on the planet. It is striking because it shows that lineages can evolve by introgressive events [7], and not just via divergence from a last common ancestor. As eukaryotes emerged from the merging of these two distantly related components, novel creatures took a central stage in the evolutionary history benefiting from novel biological properties, such as, typically, a new mode of generation of genetic variability, i.e. the general fuel for evolution, by meiosis. Such an event, which changed the course of life on Earth, has been described as an egalitarian evolutionary transition [7], because it involves the association and stabilization of elemental components with different phylogenetic origins and their transformation into a novel composite life form. Because mitochondria are considered remnants of this ancient evolutionary transition, bacterial genes are generally thought to have endowed eukaryotes with their metabolic capabilities [4–6].

A major evolutionary transition such as eukaryogenesis, giving birth to a high-level lineage, by the merging of genetic material from distantly related organisms, is usually assumed to be rare (no more than a few events per billion years). Being rare, however, does not entail that such events do not obey rules, but simply that causal rules are difficult to discover. In a recent series of original works, Nelson-Sathi *et al*. [2,3] further showed that, possibly, most major archaeal lineages likewise emerged from the merging of metabolic genes from bacteria with the genetic material of methanogenic archaea. Again, metabolic genes appeared as key elements for the birth of these novel lineages via introgressive processes, producing novel successful lines of composite beings on Earth. These findings in the archaeal domain suggest that the evolution of eukaryotes was not just one random chance event, but the outcome of a recurrent process in which metabolic genes from bacterial lineages provide genetic bases for the make-up of novel life forms. In other words, in many occasions bacterial metabolic genes were subjected to introgressive processes, and only in a limited number of occasions, these introgressions resulted in the emergence of lineages of novel beings better fitting with the adaptive demands. Therefore, the contribution of metabolic genes from bacterial lineages would be one of the rules of egalitarian evolutionary transitions. By contrast, so far, genes from archaeal [2] and from eukaryotic lineages do not seem to have contributed to the emergence of novel bacterial lineages. A major lesson from works on the origins of composite high-level phyla might be that metabolic bacterial genes are amongst the greatest and most creative evolutionary plugins of life. Acquisition of such metabolic genes likely opened or defined new niches for composite lineages, in which environmental adaptation and further habitat preference took place.

This unparalleled role of metabolic bacterial genes in shaping lineages has also been observed in several endosymbiotic events, which turned non-photosynthetic eukaryotes into photosynthetic ones [8], as well as in other fascinating introgressions [9]. Such a role is directly consistent with the frequent exchanges of metabolic genes between bacterial lineages themselves [10]. Therefore, not all genes on Earth have the same evolutionary fate and impact. Within the cellular world, metabolic bacterial genes, characterized by their amazing evolvability and their repeated crucial contribution to the make-up of lineages across all life, seem to be, by far, a most valuable source of adaptations. Why bacterial genes, rather than archaeal or eukaryotic genes, constitute such a widespread powerful evolutionary material deserves further investigation. One can only speculate. Careful analyses of genes flux, for example analyzing the turn-over of genes belonging to distinct functional categories in genomes, might show that metabolic bacterial genes persist for longer time periods than most other genes in genomes. Such a higher persistence, if observed, might explain why contributions of metabolic bacterial genes to the long-time evolution of composite lineages have been repeatedly detected. Importantly, works by Nelson-Sathi *et al.* encourages strengthening the research program on evolutionary transitions, by specifically tracking the motion and the transformative role of metabolic genes from bacterial origin in the web of life. It could provide novel and broader angles to address the issue of the origins of lineages, and the diversity of life on Earth.

## Figures and Tables

**Figure 1:**
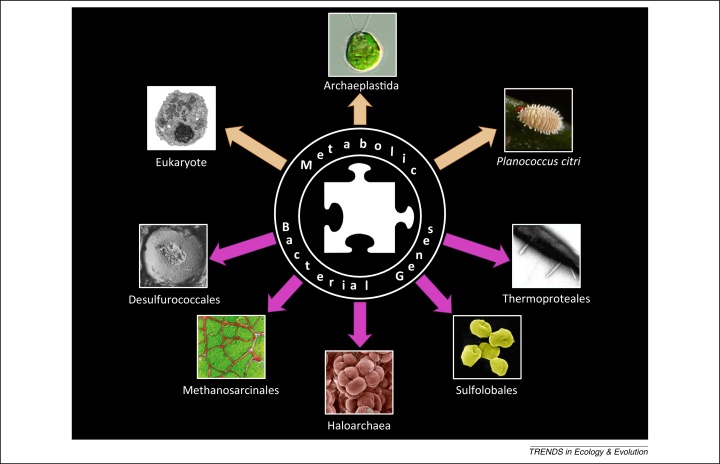
Introgression of metabolic bacterial genes: a recurrent evolutionary theme at the origin of novel composite lineages. First reports of bacterial genes contributions to the evolution of lineages were documented in eukaryotes (orange arrows), with the discoveries of eukaryogenesis, the primary chloroplastic endosymbiosis at the origins of Archaeaplastida, or of more recent endosymbioses endowing several eukaryotic lineages with additional metabolic capabilities, exemplified here by the tripartite nested mealybug symbiosis. Nelson-Sathi *et al*. [1] profoundly expanded this view, as they propose that numerous major archaeal lineages (pink arrows) also originated from the massive acquisition of bacterial genes.
